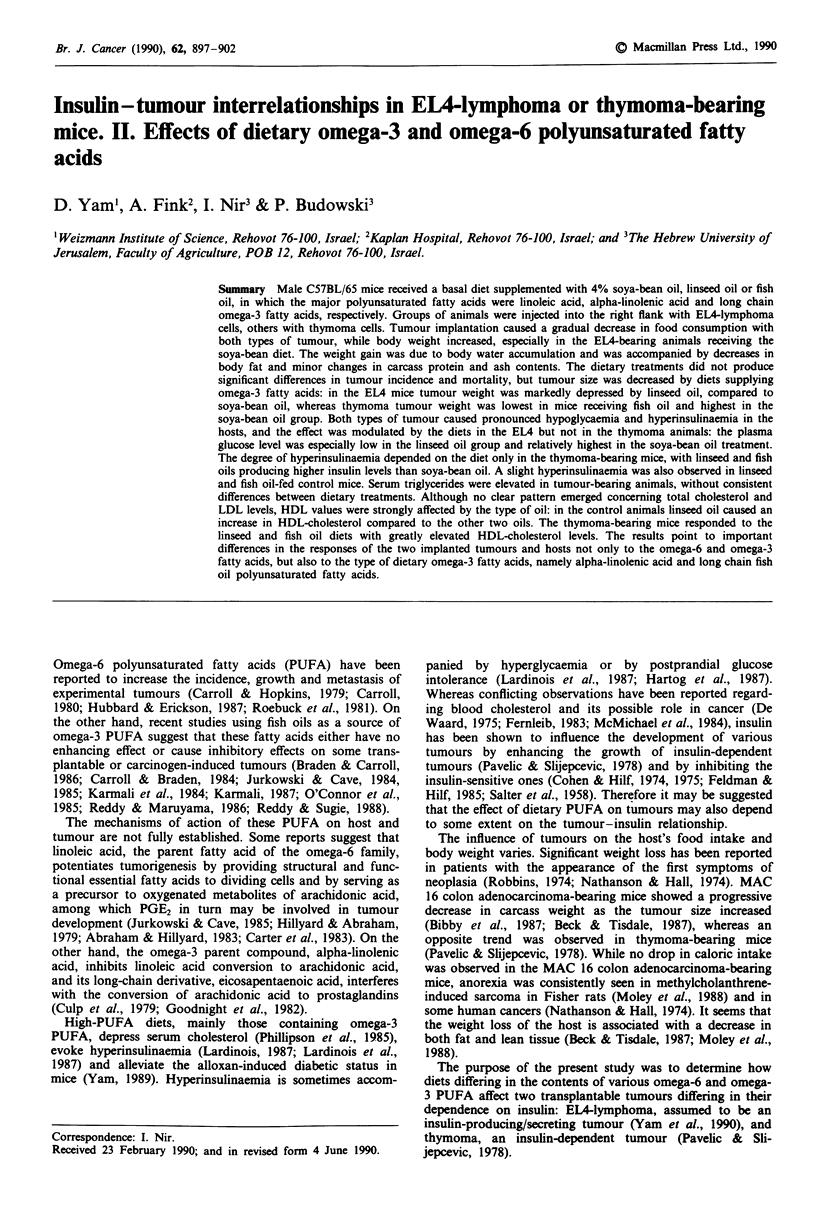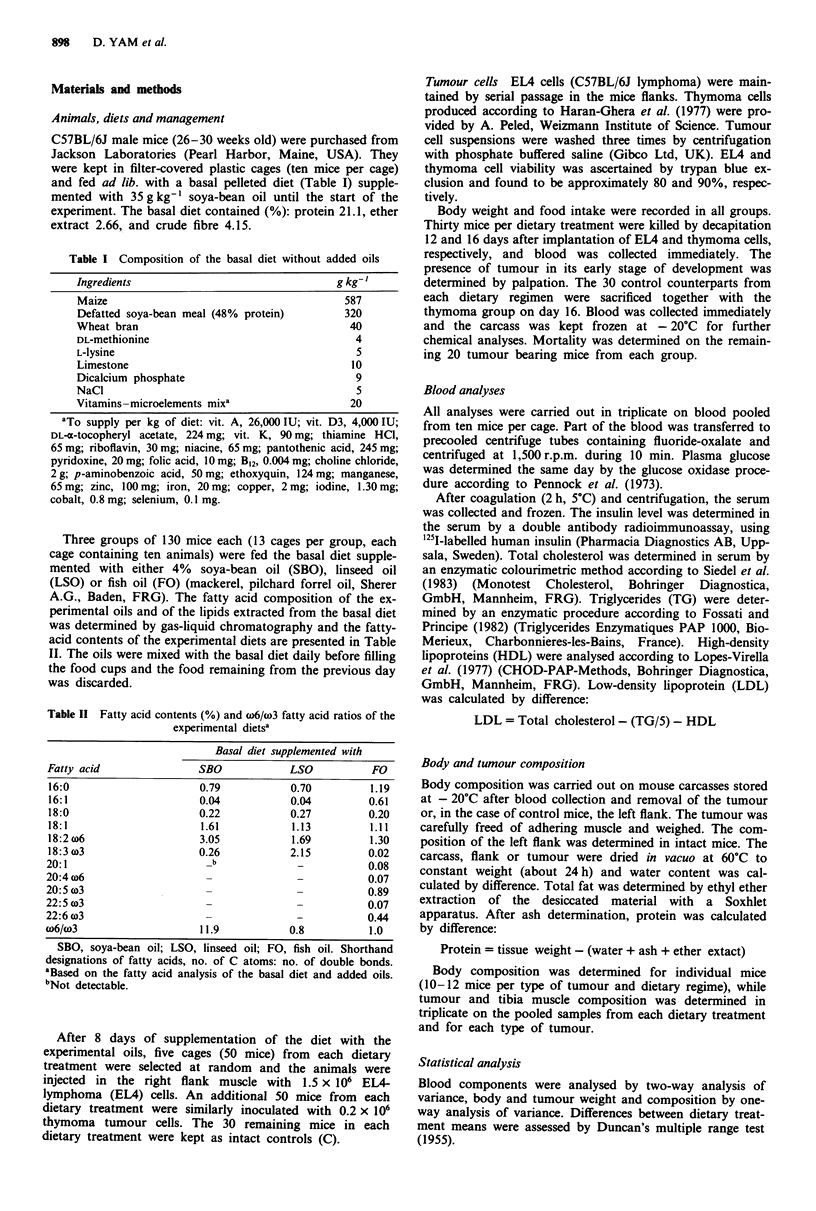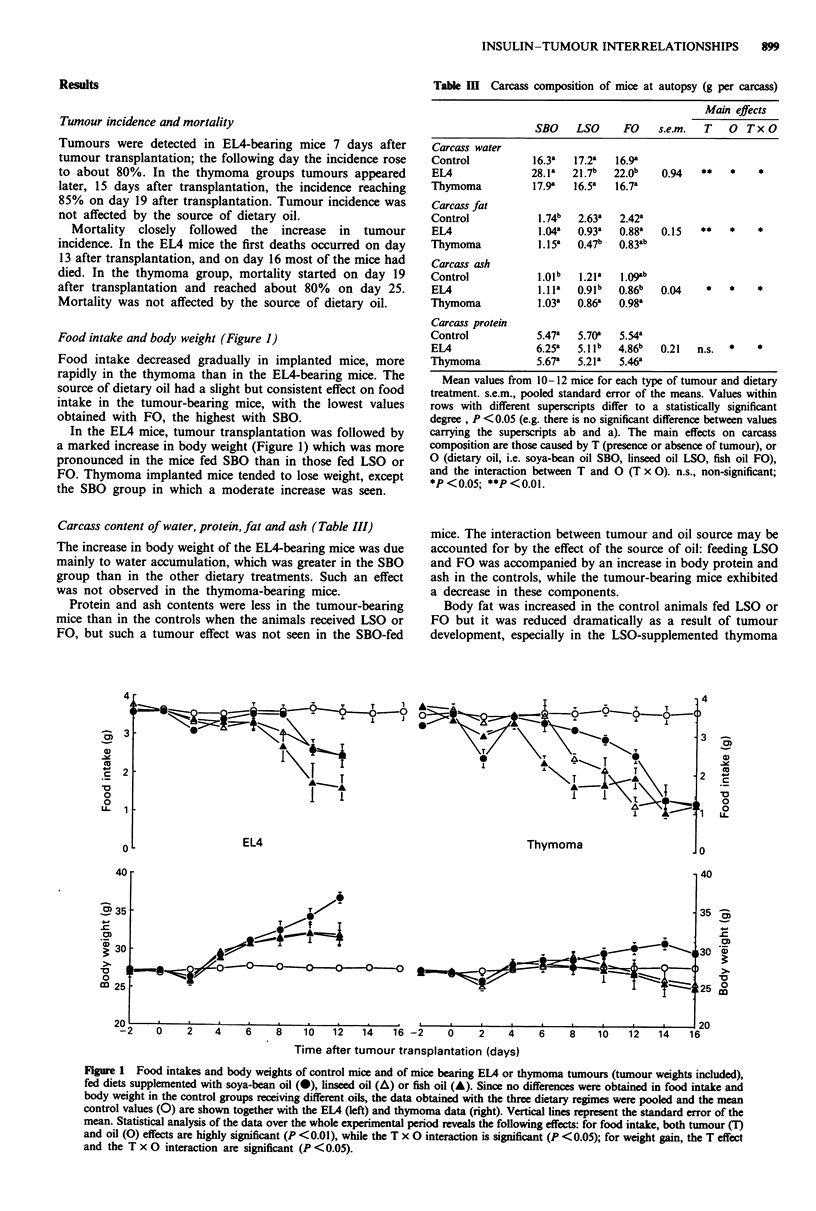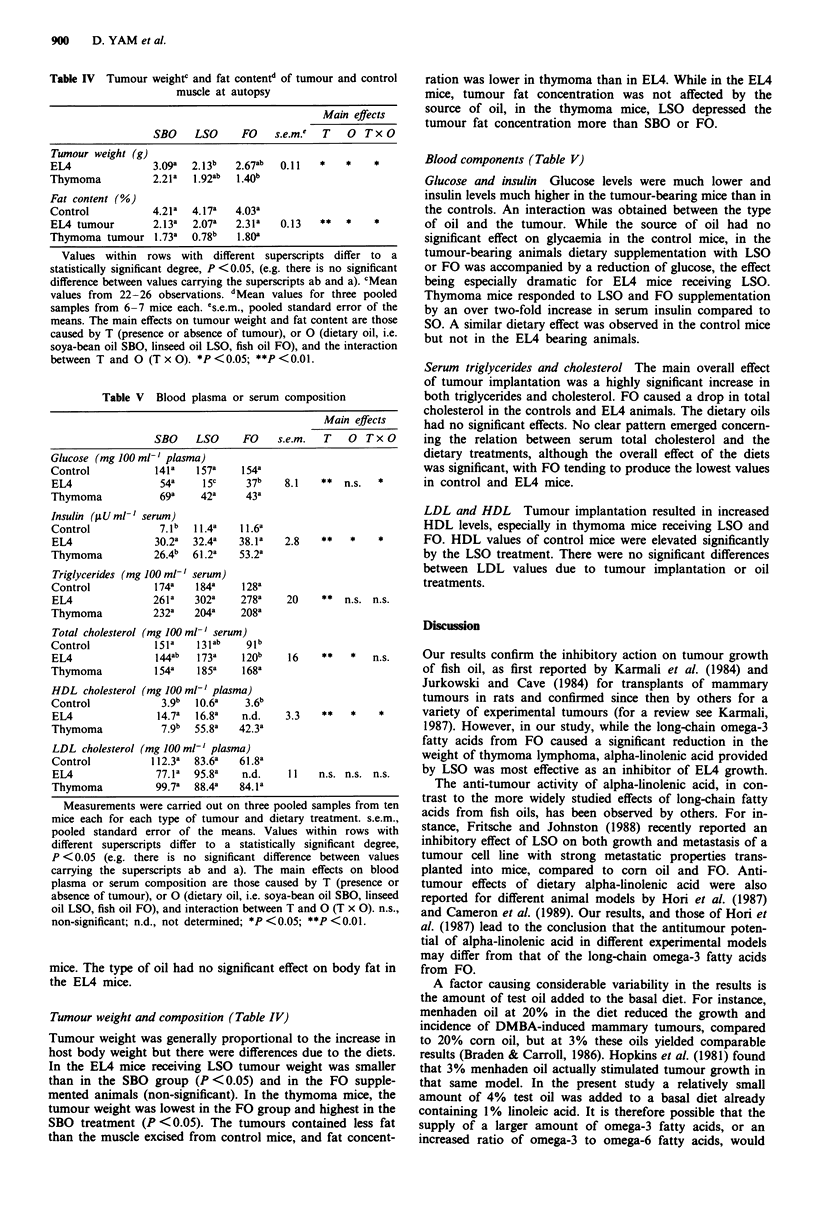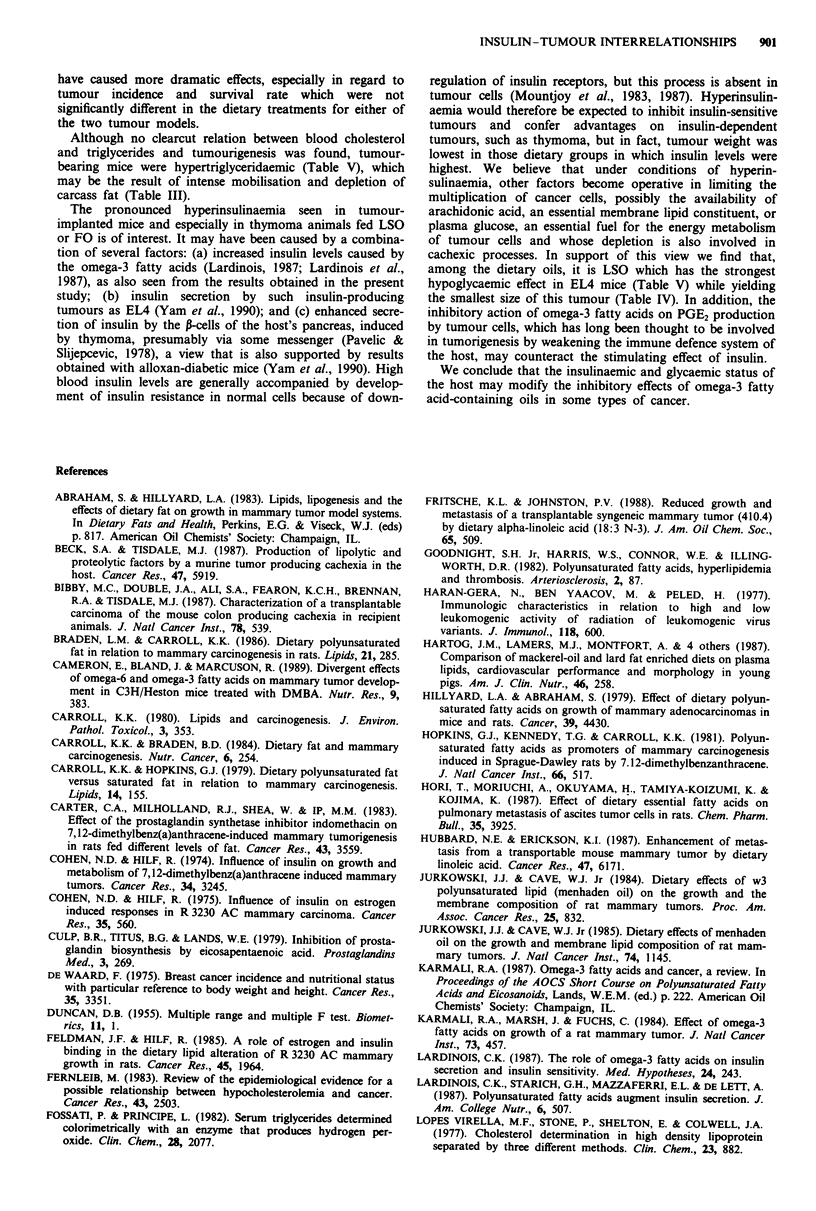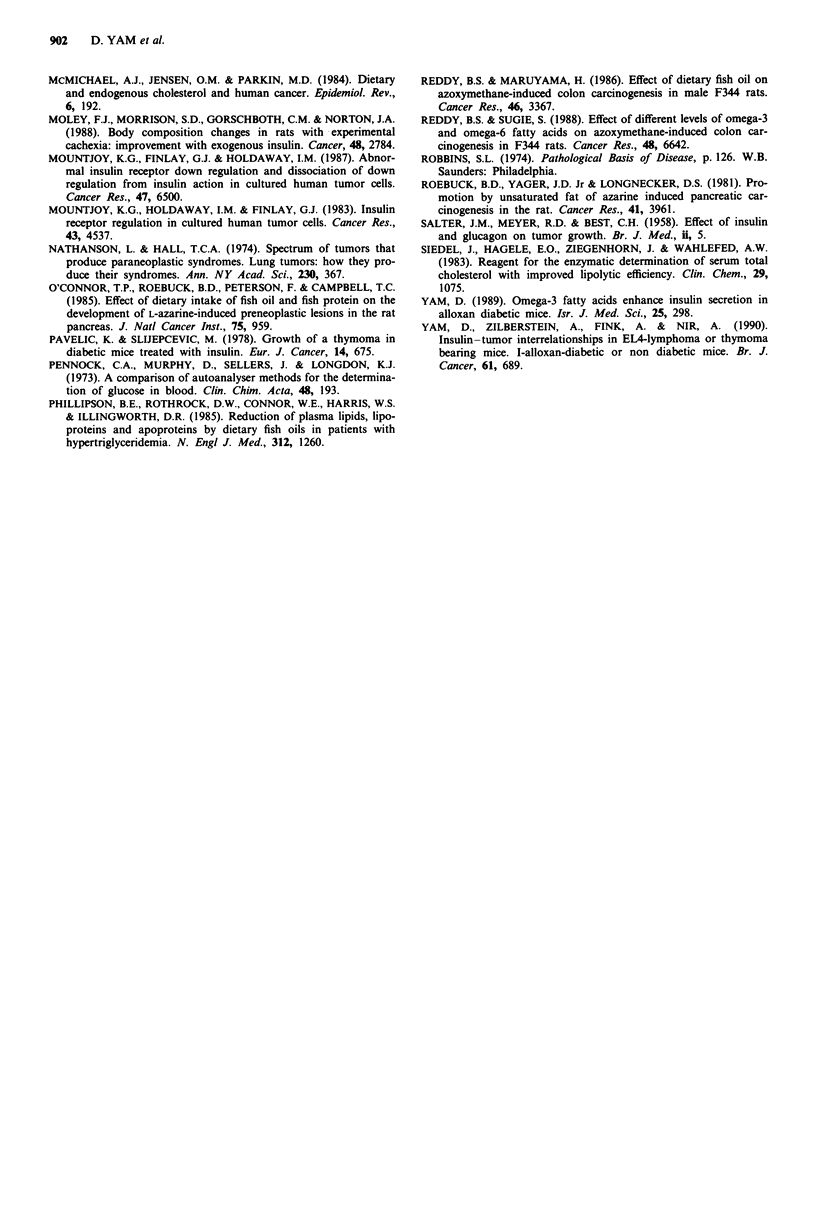# Insulin-tumour interrelationships in EL4-lymphoma or thymoma-bearing mice. II. Effects of dietary omega-3 and omega-6 polyunsaturated fatty acids.

**DOI:** 10.1038/bjc.1990.405

**Published:** 1990-12

**Authors:** D. Yam, A. Fink, I. Nir, P. Budowski

**Affiliations:** Weizmann Institute of Science, Rehovot, Israel.

## Abstract

Male C57BL/65 mice received a basal diet supplemented with 4% soya-bean oil, linseed oil or fish oil, in which the major polyunsaturated fatty acids were linoleic acid, alpha-linolenic acid and long chain omega-3 fatty acids, respectively. Groups of animals were injected into the right flank with EL4-lymphoma cells, others with thymoma cells. Tumour implantation caused a gradual decrease in food consumption with both types of tumour, while body weight increased, especially in the EL4-bearing animals receiving the soya-bean diet. The weight gain was due to body water accumulation and was accompanied by decreases in body fat and minor changes in carcass protein and ash contents. The dietary treatments did not produce significant differences in tumour incidence and mortality, but tumour size was decreased by diets supplying omega-3 fatty acids: in the EL4 mice tumour weight was markedly depressed by linseed oil, compared to soya-bean oil, whereas thymoma tumour weight was lowest in mice receiving fish oil and highest in the soya-bean oil group. Both types of tumour caused pronounced hypoglycaemia and hyperinsulinaemia in the hosts, and the effect was modulated by the diets in the EL4 but not in the thymoma animals: the plasma glucose level was especially low in the linseed oil group and relatively highest in the soya-bean oil treatment. The degree of hyperinsulinaemia depended on the diet only in the thymoma-bearing mice, with linseed and fish oils producing higher insulin levels than soya-bean oil. A slight hyperinsulinaemia was also observed in linseed and fish oil-fed control mice. Serum triglycerides were elevated in tumour-bearing animals, without consistent differences between dietary treatments. Although no clear pattern emerged concerning total cholesterol and LDL levels, HDL values were strongly affected by the type of oil: in the control animals linseed oil caused an increase in HDL-cholesterol compared to the other two oils. The thymoma-bearing mice responded to the linseed and fish oil diets with greatly elevated HDL-cholesterol levels. The results point to important differences in the responses of the two implanted tumours and hosts not only to the omega-6 and omega-3 fatty acids, but also to the type of dietary omega-3 fatty acids, namely alpha-linolenic acid and long chain fish oil polyunsaturated fatty acids.


 © Macmillan Press Ltd., 1990

### Insulin–tumour interrelationships in EL4-lymphoma or thymoma-bearing mice. II. Effects of dietary omega-3 and omega-6 polyunsaturated fatty acids

D. Yam[1], A. Fink[2], I. Nir[3] & P. Budowski[3]

[1]*Weizmann Institute of Science, Rehovot 76-100, Israel;* [2]*Kaplan Hospital, Rehovot 76-100, Israel; and* [3]*The Hebrew University of Jerusalem, Faculty of Agriculture, POB 12, Rehovot 76-100, Israel.*

**Summary** Male C57BL/65 mice received a basal diet supplemented with 4% soya-bean oil, linseed oil or fish oil, in which the major polyunsaturated fatty acids were linoleic acid, alpha-linolenic acid and long chain omega-3 fatty acids, respectively. Groups of animals were injected into the right flank with EL4-lymphoma cells, others with thymoma cells. Tumour implantation caused a gradual decrease in food consumption with both types of tumour, while body weight increased, especially in the EL4-bearing animals receiving the soya-bean diet. The weight gain was due to body water accumulation and was accompanied by decreases in body fat and minor changes in carcass protein and ash contents. The dietary treatments did not produce significant differences in tumour incidence and mortality, but tumour size was decreased by diets supplying omega-3 fatty acids: in the EL4 mice tumour weight was markedly depressed by linseed oil, compared to soya-bean oil, whereas thymoma tumour weight was lowest in mice receiving fish oil and highest in the soya-bean oil group. Both types of tumour caused pronounced hypoglycaemia and hyperinsulinaemia in the hosts, and the effect was modulated by the diets in the EL4 but not in the thymoma animals: the plasma glucose level was especially low in the linseed oil group and relatively highest in the soya-bean oil treatment. The degree of hyperinsulinaemia depended on the diet only in the thymoma-bearing mice, with linseed and fish oils producing higher insulin levels than soya-bean oil. A slight hyperinsulinaemia was also observed in linseed and fish oil-fed control mice. Serum triglycerides were elevated in tumour-bearing animals, without consistent differences between dietary treatments. Although no clear pattern emerged concerning total cholesterol and LDL levels, HDL values were strongly affected by the type of oil: in the control animals linseed oil caused an increase in HDL-cholesterol compared to the other two oils. The thymoma-bearing mice responded to the linseed and fish oil diets with greatly elevated HDL-cholesterol levels. The results point to important differences in the responses of the two implanted tumours and hosts not only to the omega-6 and omega-3 fatty acids, but also to the type of dietary omega-3 fatty acids, namely alpha-linolenic acid and long chain fish oil polyunsaturated fatty acids.

Omega-6 polyunsaturated fatty acids (PUFA) have been reported to increase the incidence, growth and metastasis of experimental tumours (Carroll & Hopkins, 1979; Carroll, 1980; Hubbard & Erickson, 1987; Roebuck *et al.*, 1981). On the other hand, recent studies using fish oils as a source of omega-3 PUFA suggest that these fatty acids either have no enhancing effect or cause inhibitory effects on some transplantable or carcinogen-induced tumours (Braden & Carroll, 1986; Carroll & Braden, 1984; Jurkowski & Cave, 1984, 1985; Karmali *et al.*, 1984; Karmali, 1987; O'Connor *et al.*, 1985; Reddy & Maruyama, 1986; Reddy & Sugie, 1988).

The mechanisms of action of these PUFA on host and tumour are not fully established. Some reports suggest that linoleic acid, the parent fatty acid of the omega-6 family, potentiates tumorigenesis by providing structural and functional essential fatty acids to dividing cells and by serving as a precursor to oxygenated metabolites of arachidonic acid, among which $PGE_2$ in turn may be involved in tumour development (Jurkowski & Cave, 1985; Hillyard & Abraham, 1979; Abraham & Hillyard, 1983; Carter *et al.*, 1983). On the other hand, the omega-3 parent compound, alpha-linolenic acid, inhibits linoleic acid conversion to arachidonic acid, and its long-chain derivative, eicosapentaenoic acid, interferes with the conversion of arachidonic acid to prostaglandins (Culp *et al.*, 1979; Goodnight *et al.*, 1982).

High-PUFA diets, mainly those containing omega-3 PUFA, depress serum cholesterol (Phillipson *et al.*, 1985), evoke hyperinsulinaemia (Lardinois, 1987; Lardinois *et al.*, 1987) and alleviate the alloxan-induced diabetic status in mice (Yam, 1989). Hyperinsulinaemia is sometimes accompanied by hyperglycaemia or by postprandial glucose intolerance (Lardinois *et al.*, 1987; Hartog *et al.*, 1987). Whereas conflicting observations have been reported regarding blood cholesterol and its possible role in cancer (De Waard, 1975; Fernleib, 1983; McMichael *et al.*, 1984), insulin has been shown to influence the development of various tumours by enhancing the growth of insulin-dependent tumours (Pavelic & Slijepcevic, 1978) and by inhibiting the insulin-sensitive ones (Cohen & Hilf, 1974, 1975; Feldman & Hilf, 1985; Salter *et al.*, 1958). Therefore it may be suggested that the effect of dietary PUFA on tumours may also depend to some extent on the tumour–insulin relationship.

The influence of tumours on the host's food intake and body weight varies. Significant weight loss has been reported in patients with the appearance of the first symptoms of neoplasia (Robbins, 1974; Nathanson & Hall, 1974). MAC 16 colon adenocarcinoma-bearing mice showed a progressive decrease in carcass weight as the tumour size increased (Bibby *et al.*, 1987; Beck & Tisdale, 1987), whereas an opposite trend was observed in thymoma-bearing mice (Pavelic & Slijepcevic, 1978). While no drop in caloric intake was observed in the MAC 16 colon adenocarcinoma-bearing mice, anorexia was consistently seen in methylcholanthrene-induced sarcoma in Fisher rats (Moley *et al.*, 1988) and in some human cancers (Nathanson & Hall, 1974). It seems that the weight loss of the host is associated with a decrease in both fat and lean tissue (Beck & Tisdale, 1987; Moley *et al.*, 1988).

The purpose of the present study was to determine how diets differing in the contents of various omega-6 and omega-3 PUFA affect two transplantable tumours differing in their dependence on insulin: EL4-lymphoma, assumed to be an insulin-producing/secreting tumour (Yam *et al.*, 1990), and thymoma, an insulin-dependent tumour (Pavelic & Slijepcevic, 1978).

Correspondence: I. Nir.
Received 23 February 1990; and in revised form 4 June 1990.

### Materials and methods

### *Animals, diets and management*

C57BL/6J male mice (26–30 weeks old) were purchased from Jackson Laboratories (Pearl Harbor, Maine, USA). They were kept in filter-covered plastic cages (ten mice per cage) and fed *ad lib.* with a basal pelleted diet (Table I) supplemented with $35\,g\,kg^{-1}$ soya-bean oil until the start of the experiment. The basal diet contained (%): protein 21.1, ether extract 2.66, and crude fibre 4.15.

**Table I** Composition of the basal diet without added oils

| *Ingredients* | $g\,kg^{-1}$ |
|---|---|
| Maize | 587 |
| Defatted soya-bean meal (48% protein) | 320 |
| Wheat bran | 40 |
| DL-methionine | 4 |
| L-lysine | 5 |
| Limestone | 10 |
| Dicalcium phosphate | 9 |
| NaCl | 5 |
| Vitamins–microelements mix[a] | 20 |

[a]To supply per kg of diet: vit. A, 26,000 IU; vit. D3, 4,000 IU; DL-α-tocopheryl acetate, 224 mg; vit. K, 90 mg; thiamine HCl, 65 mg; riboflavin, 30 mg; niacine, 65 mg; pantothenic acid, 245 mg; pyridoxine, 20 mg; folic acid, 10 mg; $B_{12}$, 0.004 mg; choline chloride, 2 g; *p*-aminobenzoic acid, 50 mg; ethoxyquin, 124 mg; manganese, 65 mg; zinc, 100 mg; iron, 20 mg; copper, 2 mg; iodine, 1.30 mg; cobalt, 0.8 mg; selenium, 0.1 mg.

Three groups of 130 mice each (13 cages per group, each cage containing ten animals) were fed the basal diet supplemented with either 4% soya-bean oil (SBO), linseed oil (LSO) or fish oil (FO) (mackerel, pilchard forrel oil, Sherer A.G., Baden, FRG). The fatty acid composition of the experimental oils and of the lipids extracted from the basal diet was determined by gas-liquid chromatography and the fatty-acid contents of the experimental diets are presented in Table II. The oils were mixed with the basal diet daily before filling the food cups and the food remaining from the previous day was discarded.

**Table II** Fatty acid contents (%) and ω6/ω3 fatty acid ratios of the experimental diets[a]

| | *Basal diet supplemented with* | | |
|---|---|---|---|
| *Fatty acid* | *SBO* | *LSO* | *FO* |
| 16:0 | 0.79 | 0.70 | 1.19 |
| 16:1 | 0.04 | 0.04 | 0.61 |
| 18:0 | 0.22 | 0.27 | 0.20 |
| 18:1 | 1.61 | 1.13 | 1.11 |
| 18:2 ω6 | 3.05 | 1.69 | 1.30 |
| 18:3 ω3 | 0.26 | 2.15 | 0.02 |
| 20:1 | –[b] | – | 0.08 |
| 20:4 ω6 | – | – | 0.07 |
| 20:5 ω3 | – | – | 0.89 |
| 22:5 ω3 | – | – | 0.07 |
| 22:6 ω3 | – | – | 0.44 |
| ω6/ω3 | 11.9 | 0.8 | 1.0 |

SBO, soya-bean oil; LSO, linseed oil; FO, fish oil. Shorthand designations of fatty acids, no. of C atoms: no. of double bonds. [a]Based on the fatty acid analysis of the basal diet and added oils. [b]Not detectable.

After 8 days of supplementation of the diet with the experimental oils, five cages (50 mice) from each dietary treatment were selected at random and the animals were injected in the right flank muscle with $1.5 \times 10^6$ EL4-lymphoma (EL4) cells. An additional 50 mice from each dietary treatment were similarly inoculated with $0.2 \times 10^6$ thymoma tumour cells. The 30 remaining mice in each dietary treatment were kept as intact controls (C).

*Tumour cells* EL4 cells (C57BL/6J lymphoma) were maintained by serial passage in the mice flanks. Thymoma cells produced according to Haran-Ghera *et al.* (1977) were provided by A. Peled, Weizmann Institute of Science. Tumour cell suspensions were washed three times by centrifugation with phosphate buffered saline (Gibco Ltd, UK). EL4 and thymoma cell viability was ascertained by trypan blue exclusion and found to be approximately 80 and 90%, respectively.

Body weight and food intake were recorded in all groups. Thirty mice per dietary treatment were killed by decapitation 12 and 16 days after implantation of EL4 and thymoma cells, respectively, and blood was collected immediately. The presence of tumour in its early stage of development was determined by palpation. The 30 control counterparts from each dietary regimen were sacrificed together with the thymoma group on day 16. Blood was collected immediately and the carcass was kept frozen at −20°C for further chemical analyses. Mortality was determined on the remaining 20 tumour bearing mice from each group.

### *Blood analyses*

All analyses were carried out in triplicate on blood pooled from ten mice per cage. Part of the blood was transferred to precooled centrifuge tubes containing fluoride-oxalate and centrifuged at 1,500 r.p.m. during 10 min. Plasma glucose was determined the same day by the glucose oxidase procedure according to Pennock *et al.* (1973).

After coagulation (2 h, 5°C) and centrifugation, the serum was collected and frozen. The insulin level was determined in the serum by a double antibody radioimmunoassay, using $^{125}$I-labelled human insulin (Pharmacia Diagnostics AB, Uppsala, Sweden). Total cholesterol was determined in serum by an enzymatic colourimetric method according to Siedel *et al.* (1983) (Monotest Cholesterol, Bohringer Diagnostica, GmbH, Mannheim, FRG). Triglycerides (TG) were determined by an enzymatic procedure according to Fossati and Principe (1982) (Triglycerides Enzymatiques PAP 1000, Bio-Merieux, Charbonnieres-les-Bains, France). High-density lipoproteins (HDL) were analysed according to Lopes-Virella *et al.* (1977) (CHOD-PAP-Methods, Bohringer Diagnostica, GmbH, Mannheim, FRG). Low-density lipoprotein (LDL) was calculated by difference:

$$LDL = \text{Total cholesterol} - (TG/5) - HDL$$

### *Body and tumour composition*

Body composition was carried out on mouse carcasses stored at −20°C after blood collection and removal of the tumour or, in the case of control mice, the left flank. The tumour was carefully freed of adhering muscle and weighed. The composition of the left flank was determined in intact mice. The carcass, flank or tumour were dried *in vacuo* at 60°C to constant weight (about 24 h) and water content was calculated by difference. Total fat was determined by ethyl ether extraction of the desiccated material with a Soxhlet apparatus. After ash determination, protein was calculated by difference:

$$\text{Protein} = \text{tissue weight} - (\text{water} + \text{ash} + \text{ether extact})$$

Body composition was determined for individual mice (10–12 mice per type of tumour and dietary regime), while tumour and tibia muscle composition was determined in triplicate on the pooled samples from each dietary treatment and for each type of tumour.

### *Statistical analysis*

Blood components were analysed by two-way analysis of variance, body and tumour weight and composition by one-way analysis of variance. Differences between dietary treatment means were assessed by Duncan's multiple range test (1955).

### Results

### *Tumour incidence and mortality*

Tumours were detected in EL4-bearing mice 7 days after tumour transplantation; the following day the incidence rose to about 80%. In the thymoma groups tumours appeared later, 15 days after transplantation, the incidence reaching 85% on day 19 after transplantation. Tumour incidence was not affected by the source of dietary oil.

Mortality closely followed the increase in tumour incidence. In the EL4 mice the first deaths occurred on day 13 after transplantation, and on day 16 most of the mice had died. In the thymoma group, mortality started on day 19 after transplantation and reached about 80% on day 25. Mortality was not affected by the source of dietary oil.

### *Food intake and body weight (Figure 1)*

Food intake decreased gradually in implanted mice, more rapidly in the thymoma than in the EL4-bearing mice. The source of dietary oil had a slight but consistent effect on food intake in the tumour-bearing mice, with the lowest values obtained with FO, the highest with SBO.

In the EL4 mice, tumour transplantation was followed by a marked increase in body weight (Figure 1) which was more pronounced in the mice fed SBO than in those fed LSO or FO. Thymoma implanted mice tended to lose weight, except the SBO group in which a moderate increase was seen.

### *Carcass content of water, protein, fat and ash (Table III)*

The increase in body weight of the EL4-bearing mice was due mainly to water accumulation, which was greater in the SBO group than in the other dietary treatments. Such an effect was not observed in the thymoma-bearing mice.

Protein and ash contents were less in the tumour-bearing mice than in the controls when the animals received LSO or FO, but such a tumour effect was not seen in the SBO-fed mice. The interaction between tumour and oil source may be accounted for by the effect of the source of oil: feeding LSO and FO was accompanied by an increase in body protein and ash in the controls, while the tumour-bearing mice exhibited a decrease in these components.

Body fat was increased in the control animals fed LSO or FO but it was reduced dramatically as a result of tumour development, especially in the LSO-supplemented thymoma

**Table III** Carcass composition of mice at autopsy (g per carcass)

| | | | | | Main effects | | |
|---|---|---|---|---|---|---|---|
| | *SBO* | *LSO* | *FO* | *s.e.m.* | *T* | *O* | *T×O* |
| *Carcass water* | | | | | | | |
| Control | $16.3^{a}$ | $17.2^{a}$ | $16.9^{a}$ | | | | |
| EL4 | $28.1^{a}$ | $21.7^{b}$ | $22.0^{b}$ | 0.94 | ** | * | * |
| Thymoma | $17.9^{a}$ | $16.5^{a}$ | $16.7^{a}$ | | | | |
| *Carcass fat* | | | | | | | |
| Control | $1.74^{b}$ | $2.63^{a}$ | $2.42^{a}$ | | | | |
| EL4 | $1.04^{a}$ | $0.93^{a}$ | $0.88^{a}$ | 0.15 | ** | * | * |
| Thymoma | $1.15^{a}$ | $0.47^{b}$ | $0.83^{ab}$ | | | | |
| *Carcass ash* | | | | | | | |
| Control | $1.01^{b}$ | $1.21^{a}$ | $1.09^{ab}$ | | | | |
| EL4 | $1.11^{a}$ | $0.91^{b}$ | $0.86^{b}$ | 0.04 | * | * | * |
| Thymoma | $1.03^{a}$ | $0.86^{a}$ | $0.98^{a}$ | | | | |
| *Carcass protein* | | | | | | | |
| Control | $5.47^{a}$ | $5.70^{a}$ | $5.54^{a}$ | | | | |
| EL4 | $6.25^{a}$ | $5.11^{b}$ | $4.86^{b}$ | 0.21 | n.s. | * | * |
| Thymoma | $5.67^{a}$ | $5.21^{a}$ | $5.46^{a}$ | | | | |

Mean values from 10–12 mice for each type of tumour and dietary treatment. s.e.m., pooled standard error of the means. Values within rows with different superscripts differ to a statistically significant degree, $P < 0.05$ (e.g. there is no significant difference between values carrying the superscripts ab and a). The main effects on carcass composition are those caused by T (presence or absence of tumour), or O (dietary oil, i.e. soya-bean oil SBO, linseed oil LSO, fish oil FO), and the interaction between T and O (T × O). n.s., non-significant; *$P < 0.05$; **$P < 0.01$.

**Figure 1** Food intakes and body weights of control mice and of mice bearing EL4 or thymoma tumours (tumour weights included), fed diets supplemented with soya-bean oil (●), linseed oil (△) or fish oil (▲). Since no differences were obtained in food intake and body weight in the control groups receiving different oils, the data obtained with the three dietary regimes were pooled and the mean control values (○) are shown together with the EL4 (left) and thymoma data (right). Vertical lines represent the standard error of the mean. Statistical analysis of the data over the whole experimental period reveals the following effects: for food intake, both tumour (T) and oil (O) effects are highly significant ($P < 0.01$), while the T × O interaction is significant ($P < 0.05$); for weight gain, the T effect and the T × O interaction are significant ($P < 0.05$).

**Table IV** Tumour weight[c] and fat content[d] of tumour and control muscle at autopsy

| | SBO | LSO | FO | s.e.m.[e] | Main effects T | O | T×O |
|---|---|---|---|---|---|---|---|
| *Tumour weight (g)* | | | | | | | |
| EL4 | 3.09[a] | 2.13[b] | 2.67[ab] | 0.11 | * | * | * |
| Thymoma | 2.21[a] | 1.92[ab] | 1.40[b] | | | | |
| *Fat content (%)* | | | | | | | |
| Control | 4.21[a] | 4.17[a] | 4.03[a] | | | | |
| EL4 tumour | 2.13[a] | 2.07[a] | 2.31[a] | 0.13 | ** | * | * |
| Thymoma tumour | 1.73[a] | 0.78[b] | 1.80[a] | | | | |

Values within rows with different superscripts differ to a statistically significant degree, $P<0.05$, (e.g. there is no significant difference between values carrying the superscripts ab and a). [c]Mean values from 22–26 observations. [d]Mean values for three pooled samples from 6–7 mice each. [e]s.e.m., pooled standard error of the means. The main effects on tumour weight and fat content are those caused by T (presence or absence of tumour), or O (dietary oil, i.e. soya-bean oil SBO, linseed oil LSO, fish oil FO), and the interaction between T and O (T × O). $^*P<0.05$; $^{**}P<0.01$.

**Table V** Blood plasma or serum composition

| | SBO | LSO | FO | s.e.m. | Main effects T | O | T×O |
|---|---|---|---|---|---|---|---|
| *Glucose (mg 100 ml$^{-1}$ plasma)* | | | | | | | |
| Control | 141[a] | 157[a] | 154[a] | | | | |
| EL4 | 54[a] | 15[c] | 37[b] | 8.1 | ** | n.s. | * |
| Thymoma | 69[a] | 42[a] | 43[a] | | | | |
| *Insulin (μU ml$^{-1}$ serum)* | | | | | | | |
| Control | 7.1[b] | 11.4[a] | 11.6[a] | | | | |
| EL4 | 30.2[a] | 32.4[a] | 38.1[a] | 2.8 | ** | * | * |
| Thymoma | 26.4[b] | 61.2[a] | 53.2[a] | | | | |
| *Triglycerides (mg 100 ml$^{-1}$ serum)* | | | | | | | |
| Control | 174[a] | 184[a] | 128[a] | | | | |
| EL4 | 261[a] | 302[a] | 278[a] | 20 | ** | n.s. | n.s. |
| Thymoma | 232[a] | 204[a] | 208[a] | | | | |
| *Total cholesterol (mg 100 ml$^{-1}$ serum)* | | | | | | | |
| Control | 151[a] | 131[ab] | 91[b] | | | | |
| EL4 | 144[ab] | 173[a] | 120[b] | 16 | ** | * | n.s. |
| Thymoma | 154[a] | 185[a] | 168[a] | | | | |
| *HDL cholesterol (mg 100 ml$^{-1}$ plasma)* | | | | | | | |
| Control | 3.9[b] | 10.6[a] | 3.6[b] | | | | |
| EL4 | 14.7[a] | 16.8[a] | n.d. | 3.3 | ** | * | * |
| Thymoma | 7.9[b] | 55.8[a] | 42.3[a] | | | | |
| *LDL cholesterol (mg 100 ml$^{-1}$ plasma)* | | | | | | | |
| Control | 112.3[a] | 83.6[a] | 61.8[a] | | | | |
| EL4 | 77.1[a] | 95.8[a] | n.d. | 11 | n.s. | n.s. | n.s. |
| Thymoma | 99.7[a] | 88.4[a] | 84.1[a] | | | | |

Measurements were carried out on three pooled samples from ten mice each for each type of tumour and dietary treatment. s.e.m., pooled standard error of the means. Values within rows with different superscripts differ to a statistically significant degree, $P<0.05$ (e.g. there is no significant difference between values carrying the superscripts ab and a). The main effects on blood plasma or serum composition are those caused by T (presence or absence of tumour), or O (dietary oil, i.e. soya-bean oil SBO, linseed oil LSO, fish oil FO), and interaction between T and O (T × O). n.s., non-significant; n.d., not determined; $^*P<0.05$; $^{**}P<0.01$.

mice. The type of oil had no significant effect on body fat in the EL4 mice.

*Tumour weight and composition (Table IV)*

Tumour weight was generally proportional to the increase in host body weight but there were differences due to the diets. In the EL4 mice receiving LSO tumour weight was smaller than in the SBO group ($P<0.05$) and in the FO supplemented animals (non-significant). In the thymoma mice, the tumour weight was lowest in the FO group and highest in the SBO treatment ($P<0.05$). The tumours contained less fat than the muscle excised from control mice, and fat concentration was lower in thymoma than in EL4. While in the EL4 mice, tumour fat concentration was not affected by the source of oil, in the thymoma mice, LSO depressed the tumour fat concentration more than SBO or FO.

*Blood components (Table V)*

*Glucose and insulin* Glucose levels were much lower and insulin levels much higher in the tumour-bearing mice than in the controls. An interaction was obtained between the type of oil and the tumour. While the source of oil had no significant effect on glycaemia in the control mice, in the tumour-bearing animals dietary supplementation with LSO or FO was accompanied by a reduction of glucose, the effect being especially dramatic for EL4 mice receiving LSO. Thymoma mice responded to LSO and FO supplementation by an over two-fold increase in serum insulin compared to SO. A similar dietary effect was observed in the control mice but not in the EL4 bearing animals.

*Serum triglycerides and cholesterol* The main overall effect of tumour implantation was a highly significant increase in both triglycerides and cholesterol. FO caused a drop in total cholesterol in the controls and EL4 animals. The dietary oils had no significant effects. No clear pattern emerged concerning the relation between serum total cholesterol and the dietary treatments, although the overall effect of the diets was significant, with FO tending to produce the lowest values in control and EL4 mice.

*LDL and HDL* Tumour implantation resulted in increased HDL levels, especially in thymoma mice receiving LSO and FO. HDL values of control mice were elevated significantly by the LSO treatment. There were no significant differences between LDL values due to tumour implantation or oil treatments.

### Discussion

Our results confirm the inhibitory action on tumour growth of fish oil, as first reported by Karmali *et al.* (1984) and Jurkowski and Cave (1984) for transplants of mammary tumours in rats and confirmed since then by others for a variety of experimental tumours (for a review see Karmali, 1987). However, in our study, while the long-chain omega-3 fatty acids from FO caused a significant reduction in the weight of thymoma lymphoma, alpha-linolenic acid provided by LSO was most effective as an inhibitor of EL4 growth.

The anti-tumour activity of alpha-linolenic acid, in contrast to the more widely studied effects of long-chain fatty acids from fish oils, has been observed by others. For instance, Fritsche and Johnston (1988) recently reported an inhibitory effect of LSO on both growth and metastasis of a tumour cell line with strong metastatic properties transplanted into mice, compared to corn oil and FO. Anti-tumour effects of dietary alpha-linolenic acid were also reported for different animal models by Hori *et al.* (1987) and Cameron *et al.* (1989). Our results, and those of Hori *et al.* (1987) lead to the conclusion that the antitumour potential of alpha-linolenic acid in different experimental models may differ from that of the long-chain omega-3 fatty acids from FO.

A factor causing considerable variability in the results is the amount of test oil added to the basal diet. For instance, menhaden oil at 20% in the diet reduced the growth and incidence of DMBA-induced mammary tumours, compared to 20% corn oil, but at 3% these oils yielded comparable results (Braden & Carroll, 1986). Hopkins *et al.* (1981) found that 3% menhaden oil actually stimulated tumour growth in that same model. In the present study a relatively small amount of 4% test oil was added to a basal diet already containing 1% linoleic acid. It is therefore possible that the supply of a larger amount of omega-3 fatty acids, or an increased ratio of omega-3 to omega-6 fatty acids, would have caused more dramatic effects, especially in regard to tumour incidence and survival rate which were not significantly different in the dietary treatments for either of the two tumour models.

Although no clearcut relation between blood cholesterol and triglycerides and tumourigenesis was found, tumour-bearing mice were hypertriglyceridaemic (Table V), which may be the result of intense mobilisation and depletion of carcass fat (Table III).

The pronounced hyperinsulinaemia seen in tumour-implanted mice and especially in thymoma animals fed LSO or FO is of interest. It may have been caused by a combination of several factors: (a) increased insulin levels caused by the omega-3 fatty acids (Lardinois, 1987; Lardinois *et al.*, 1987), as also seen from the results obtained in the present study; (b) insulin secretion by such insulin-producing tumours as EL4 (Yam *et al.*, 1990); and (c) enhanced secretion of insulin by the $\beta$-cells of the host's pancreas, induced by thymoma, presumably via some messenger (Pavelic & Slijepcevic, 1978), a view that is also supported by results obtained with alloxan-diabetic mice (Yam *et al.*, 1990). High blood insulin levels are generally accompanied by development of insulin resistance in normal cells because of down-regulation of insulin receptors, but this process is absent in tumour cells (Mountjoy *et al.*, 1983, 1987). Hyperinsulinaemia would therefore be expected to inhibit insulin-sensitive tumours and confer advantages on insulin-dependent tumours, such as thymoma, but in fact, tumour weight was lowest in those dietary groups in which insulin levels were highest. We believe that under conditions of hyperinsulinaemia, other factors become operative in limiting the multiplication of cancer cells, possibly the availability of arachidonic acid, an essential membrane lipid constituent, or plasma glucose, an essential fuel for the energy metabolism of tumour cells and whose depletion is also involved in cachexic processes. In support of this view we find that, among the dietary oils, it is LSO which has the strongest hypoglycaemic effect in EL4 mice (Table V) while yielding the smallest size of this tumour (Table IV). In addition, the inhibitory action of omega-3 fatty acids on $PGE_2$ production by tumour cells, which has long been thought to be involved in tumorigenesis by weakening the immune defence system of the host, may counteract the stimulating effect of insulin.

We conclude that the insulinaemic and glycaemic status of the host may modify the inhibitory effects of omega-3 fatty acid-containing oils in some types of cancer.

### References

ABRAHAM, S. & HILLYARD, L.A. (1983). Lipids, lipogenesis and the effects of dietary fat on growth in mammary tumor model systems. In *Dietary Fats and Health*, Perkins, E.G. & Viseck, W.J. (eds) p. 817. American Oil Chemists' Society: Champaign, IL.

BECK, S.A. & TISDALE, M.J. (1987). Production of lipolytic and proteolytic factors by a murine tumor producing cachexia in the host. *Cancer Res.*, **47**, 5919.

BIBBY, M.C., DOUBLE, J.A., ALI, S.A., FEARON, K.C.H., BRENNAN, R.A. & TISDALE, M.J. (1987). Characterization of a transplantable carcinoma of the mouse colon producing cachexia in recipient animals. *J. Natl Cancer Inst.*, **78**, 539.

BRADEN, L.M. & CARROLL, K.K. (1986). Dietary polyunsaturated fat in relation to mammary carcinogenesis in rats. *Lipids*, **21**, 285.

CAMERON, E., BLAND, J. & MARCUSON, R. (1989). Divergent effects of omega-6 and omega-3 fatty acids on mammary tumor development in C3H/Heston mice treated with DMBA. *Nutr. Res.*, **9**, 383.

CARROLL, K.K. (1980). Lipids and carcinogenesis. *J. Environ. Pathol. Toxicol.*, **3**, 353.

CARROLL, K.K. & BRADEN, B.D. (1984). Dietary fat and mammary carcinogenesis. *Nutr. Cancer*, **6**, 254.

CARROLL, K.K. & HOPKINS, G.J. (1979). Dietary polyunsaturated fat versus saturated fat in relation to mammary carcinogenesis. *Lipids*, **14**, 155.

CARTER, C.A., MILHOLLAND, R.J., SHEA, W. & IP, M.M. (1983). Effect of the prostaglandin synthetase inhibitor indomethacin on 7,12-dimethylbenz(a)anthracene-induced mammary tumorigenesis in rats fed different levels of fat. *Cancer Res.*, **43**, 3559.

COHEN, N.D. & HILF, R. (1974). Influence of insulin on growth and metabolism of 7,12-dimethylbenz(a)anthracene induced mammary tumors. *Cancer Res.*, **34**, 3245.

COHEN, N.D. & HILF, R. (1975). Influence of insulin on estrogen induced responses in R 3230 AC mammary carcinoma. *Cancer Res.*, **35**, 560.

CULP, B.R., TITUS, B.G. & LANDS, W.E. (1979). Inhibition of prostaglandin biosynthesis by eicosapentaenoic acid. *Prostaglandins Med.*, **3**, 269.

DE WAARD, F. (1975). Breast cancer incidence and nutritional status with particular reference to body weight and height. *Cancer Res.*, **35**, 3351.

DUNCAN, D.B. (1955). Multiple range and multiple F test. *Biometrics*, **11**, 1.

FELDMAN, J.F. & HILF, R. (1985). A role of estrogen and insulin binding in the dietary lipid alteration of R 3230 AC mammary growth in rats. *Cancer Res.*, **45**, 1964.

FERNLEIB, M. (1983). Review of the epidemiological evidence for a possible relationship between hypocholesterolemia and cancer. *Cancer Res.*, **43**, 2503.

FOSSATI, P. & PRINCIPE, L. (1982). Serum triglycerides determined colorimetrically with an enzyme that produces hydrogen peroxide. *Clin. Chem.*, **28**, 2077.

FRITSCHE, K.L. & JOHNSTON, P.V. (1988). Reduced growth and metastasis of a transplantable syngeneic mammary tumor (410.4) by dietary alpha-linoleic acid (18:3 N-3). *J. Am. Oil Chem. Soc.*, **65**, 509.

GOODNIGHT, S.H. Jr, HARRIS, W.S., CONNOR, W.E. & ILLINGWORTH, D.R. (1982). Polyunsaturated fatty acids, hyperlipidemia and thrombosis. *Arteriosclerosis*, **2**, 87.

HARAN-GERA, N., BEN YAACOV, M. & PELED, H. (1977). Immunologic characteristics in relation to high and low leukomogenic activity of radiation of leukomogenic virus variants. *J. Immunol.*, **118**, 600.

HARTOG, J.M., LAMERS, M.J., MONTFORT, A. & 4 others (1987). Comparison of mackerel-oil and lard fat enriched diets on plasma lipids, cardiovascular performance and morphology in young pigs. *Am. J. Clin. Nutr.*, **46**, 258.

HILLYARD, L.A. & ABRAHAM, S. (1979). Effect of dietary polyunsaturated fatty acids on growth of mammary adenocarcinomas in mice and rats. *Cancer*, **39**, 4430.

HOPKINS, G.J., KENNEDY, T.G. & CARROLL, K.K. (1981). Polyunsaturated fatty acids as promoters of mammary carcinogenesis induced in Sprague-Dawley rats by 7.12-dimethylbenzanthracene. *J. Natl Cancer Inst.*, **66**, 517.

HORI, T., MORIUCHI, A., OKUYAMA, H., TAMIYA-KOIZUMI, K. & KOJIMA, K. (1987). Effect of dietary essential fatty acids on pulmonary metastasis of ascites tumor cells in rats. *Chem. Pharm. Bull.*, **35**, 3925.

HUBBARD, N.E. & ERICKSON, K.I. (1987). Enhancement of metastasis from a transportable mouse mammary tumor by dietary linoleic acid. *Cancer Res.*, **47**, 6171.

JURKOWSKI, J.J. & CAVE, W.J. Jr (1984). Dietary effects of w3 polyunsaturated lipid (menhaden oil) on the growth and the membrane composition of rat mammary tumors. *Proc. Am. Assoc. Cancer Res.*, **25**, 832.

JURKOWSKI, J.J. & CAVE, W.J. Jr (1985). Dietary effects of menhaden oil on the growth and membrane lipid composition of rat mammary tumors. *J. Natl Cancer Inst.*, **74**, 1145.

KARMALI, R.A. (1987). Omega-3 fatty acids and cancer, a review. In *Proceedings of the AOCS Short Course on Polyunsaturated Fatty Acids and Eicosanoids*, Lands, W.E.M. (ed.) p. 222. American Oil Chemists' Society: Champaign, IL.

KARMALI, R.A., MARSH, J. & FUCHS, C. (1984). Effect of omega-3 fatty acids on growth of a rat mammary tumor. *J. Natl Cancer Inst.*, **73**, 457.

LARDINOIS, C.K. (1987). The role of omega-3 fatty acids on insulin secretion and insulin sensitivity. *Med. Hypotheses*, **24**, 243.

LARDINOIS, C.K., STARICH, G.H., MAZZAFERRI, E.L. & DE LETT, A. (1987). Polyunsaturated fatty acids augment insulin secretion. *J. Am. College Nutr.*, **6**, 507.

LOPES VIRELLA, M.F., STONE, P., SHELTON, E. & COLWELL, J.A. (1977). Cholesterol determination in high density lipoprotein separated by three different methods. *Clin. Chem.*, **23**, 882.

MCMICHAEL, A.J., JENSEN, O.M. & PARKIN, M.D. (1984). Dietary and endogenous cholesterol and human cancer. *Epidemiol. Rev.*, **6**, 192.

MOLEY, F.J., MORRISON, S.D., GORSCHBOTH, C.M. & NORTON, J.A. (1988). Body composition changes in rats with experimental cachexia: improvement with exogenous insulin. *Cancer*, **48**, 2784.

MOUNTJOY, K.G., FINLAY, G.J. & HOLDAWAY, I.M. (1987). Abnormal insulin receptor down regulation and dissociation of down regulation from insulin action in cultured human tumor cells. *Cancer Res.*, **47**, 6500.

MOUNTJOY, K.G., HOLDAWAY, I.M. & FINLAY, G.J. (1983). Insulin receptor regulation in cultured human tumor cells. *Cancer Res.*, **43**, 4537.

NATHANSON, L. & HALL, T.C.A. (1974). Spectrum of tumors that produce paraneoplastic syndromes. Lung tumors: how they produce their syndromes. *Ann. NY Acad. Sci.*, **230**, 367.

O'CONNOR, T.P., ROEBUCK, B.D., PETERSON, F. & CAMPBELL, T.C. (1985). Effect of dietary intake of fish oil and fish protein on the development of L-azarine-induced preneoplastic lesions in the rat pancreas. *J. Natl Cancer Inst.*, **75**, 959.

PAVELIC, K. & SLIJEPCEVIC, M. (1978). Growth of a thymoma in diabetic mice treated with insulin. *Eur. J. Cancer*, **14**, 675.

PENNOCK, C.A., MURPHY, D., SELLERS, J. & LONGDON, K.J. (1973). A comparison of autoanalyser methods for the determination of glucose in blood. *Clin. Chim. Acta*, **48**, 193.

PHILLIPSON, B.E., ROTHROCK, D.W., CONNOR, W.E., HARRIS, W.S. & ILLINGWORTH, D.R. (1985). Reduction of plasma lipids, lipoproteins and apoproteins by dietary fish oils in patients with hypertriglyceridemia. *N. Engl J. Med.*, **312**, 1260.

REDDY, B.S. & MARUYAMA, H. (1986). Effect of dietary fish oil on azoxymethane-induced colon carcinogenesis in male F344 rats. *Cancer Res.*, **46**, 3367.

REDDY, B.S. & SUGIE, S. (1988). Effect of different levels of omega-3 and omega-6 fatty acids on azoxymethane-induced colon carcinogenesis in F344 rats. *Cancer Res.*, **48**, 6642.

ROBBINS, S.L. (1974). *Pathological Basis of Disease*, p. 126. W.B. Saunders: Philadelphia.

ROEBUCK, B.D., YAGER, J.D. Jr & LONGNECKER, D.S. (1981). Promotion by unsaturated fat of azarine induced pancreatic carcinogenesis in the rat. *Cancer Res.*, **41**, 3961.

SALTER, J.M., MEYER, R.D. & BEST, C.H. (1958). Effect of insulin and glucagon on tumor growth. *Br. J. Med.*, **ii**, 5.

SIEDEL, J., HAGELE, E.O., ZIEGENHORN, J. & WAHLEFED, A.W. (1983). Reagent for the enzymatic determination of serum total cholesterol with improved lipolytic efficiency. *Clin. Chem.*, **29**, 1075.

YAM, D. (1989). Omega-3 fatty acids enhance insulin secretion in alloxan diabetic mice. *Isr. J. Med. Sci.*, **25**, 298.

YAM, D., ZILBERSTEIN, A., FINK, A. & NIR, A. (1990). Insulin–tumor interrelationships in EL4-lymphoma or thymoma bearing mice. I-alloxan-diabetic or non diabetic mice. *Br. J. Cancer*, **61**, 689.